# 

**Published:** 1897-04-17

**Authors:** 


					i nhi iiuui  AOOuiiOTiiL x r-urs.ui, mciciuic '* "*> *
CADBURY'S ft w. CADBURY'S
COCOA
" The standard of highest purity at present -S. Ml* NO alkalies used.
attainable. ?Lancet,
COCOA
Lasqo if Wes tern INFtRMAnr
No. 55 . Yol. XXII. [_ilegpeadpe^] Saturday,
April 17th, 1897.
[SabscripUonTerms,] pE)ICE TWOPENCE.

				

